# Reliability of forearm oxygen uptake during handgrip exercise: assessment by ultrasonography and venous blood gas

**DOI:** 10.14814/phy2.13696

**Published:** 2018-05-20

**Authors:** Stian K. Nyberg, Ole Kristian Berg, Jan Helgerud, Eivind Wang

**Affiliations:** ^1^ Department of Circulation and Medical Imaging Faculty of Medicine Norwegian University of Science and Technology Trondheim Norway; ^2^ Faculty of Health and Social Sciences Molde University College Molde Norway; ^3^ Department of Internal Medicine University of Utah Salt Lake City Utah

**Keywords:** Blood flow, oxygen extraction, small muscle mass, test–retest, $\dot{V}$O_2_

## Abstract

Assessment of forearm oxygen uptake ($\dot{V}$O_2_) during handgrip exercise is a keenly investigated concept for observing small muscle mass metabolism. Although a combination of Doppler ultrasound measurements of brachial artery blood flow ($\dot{Q}$) and blood gas drawn from a deep forearm vein has been utilized to calculate forearm $\dot{V}$O_2_ for more than two decades, the applicability of this experimental design may benefit from a thorough evaluation of its reliability during graded exercise. Therefore, we evaluated the reliability of this technique during incremental handgrip exercise in ten healthy young (24 ± 3(SD) years.) males. $\dot{V}$O_2_ and work rate (WR) exhibited a linear relationship (1.0 W: 43.8 ± 10.1 mL·min^−1^; 1.5 W: 53.8 ± 14.1 mL·min^−1^; 2.0 W: 63.4 ± 16.3 mL·min^−1^; 2.5 W: 72.2 ± 17.6 mL·min^−1^; 3.0 W: 79.2 ± 18.6 mL·min^−1^; *r* = 0.65, *P* < 0.01). In turn, $\dot{V}$O_2_ was strongly associated with $\dot{Q}$ (1.0 W: 359 ± 86 mL·min^−1^; 1.5 W: 431 ± 112 mL·min^−1^; 2.0 W: 490 ± 123 mL·min^−1^; 2.5 W: 556 ± 112 mL·min^−1^; 3.0 W: 622 ± 131 mL·min^−1^; *r* = 0.96; *P* < 0.01), whereas arteriovenous oxygen difference (a‐vO_2diff_) remained constant following all WRs (123 ± 11–130 ± 10 mL·L^−1^). Average $\dot{V}$O_2_ test–retest difference was −0.4 mL·min^−1^ with ±2SD limits of agreement (LOA) of 8.4 and −9.2 mL·min^−1^, respectively, whereas coefficients of variation (CVs) ranged from 4–7%. Accordingly, test–retest $\dot{Q}$ difference was 11.9 mL·min^−1^ (LOA: 84.1 mL·min^−1^; −60.4 mL·min^−1^) with CVs between 4 and 7%. Test–retest difference for a‐vO_2diff_ was −0.28 mL·dL^−1^ (LOA: 1.26mL·dL ^−1^; −1.82 mL·dL^−1^) with 3–5% CVs. In conclusion, our results revealed that forearm $\dot{V}$O_2_ determination by Doppler ultrasound and direct venous sampling is linearly related to WR, and a reliable experimental design across a range of exercise intensities.

## Introduction

The handgrip exercise model has been extensively used in the investigation of small muscle mass metabolism for half a century (Greenwood et al. 1965; Seaman et al. 1973; Hughson et al. 1996; Broxterman et al. 2015). The forearm provides a relevant approach to investigate the matching of oxygen supply to demand when oxygen transport is unrestricted by central factors, it is also of interest because of its essential part of everyday life. In light of this, hand grip exercise has also been extensively used to offer key insight into the peripheral impact of cardiac, vascular, or pulmonary diseases (Shoemaker et al. 1999; Barrett‐O'Keefe et al. 2014; Haarmann et al. 2016). Doppler ultrasound technology, in conjunction with blood gases from veins draining the forearm, has been established as a prime, minimally invasive, experimental design to calculate oxygen uptake ($\dot{V}$O_2_) based on the Fick principle (Hughson et al. 1996; Shoemaker et al. 1999; Lee et al. 2000; MacDonald et al. 2001; Van Beekvelt et al. 2001; Crecelius et al. 2013). However, despite extensive utilization, the reliability of this experimental design has not been thoroughly investigated to date. The reliability of a method refers to the reproducibility of test values, and is important for evaluating estimates of change in a variable of an experimental study (Hopkins 2000). Hence, it remains unclear with what confidence data can be interpreted.

$\dot{V}$O_2_ is largely dictated by blood flow ($\dot{Q}$) hindrance from muscle contractions during vigorous handgrip exercise (Nyberg et al. 2017). Doppler ultrasound is a continuously advancing technology for observing the dynamic $\dot{Q}$ behavior during the entire contraction–relaxation cycle, and tailored for this purpose through detection of blood velocity and direction in combination with diameter determination (Reneman and Hoeks 2000; Harvey et al. 2002). Of note, these data are not available when dye‐ / thermodilution or isotope clearance methods are utilized to investigate $\dot{Q}$ (Radegran 1999). In particular, the ability to simultaneously measure pulse wave Doppler and B‐mode images, saved in one file for subsequent analysis, has made acquisition of $\dot{Q}$ less prone to errors in placing sample volume and angle corrections (Ekroll et al. 2014). However, even an early version of the Doppler ultrasound technology, applied during handgrip exercise, was concluded to be reliable when used in isolation (Shoemaker et al. 1996).

The forearm also provides relatively easy access to the venous blood vessels draining the exercising musculature (Holling 1939; Mottram 1955). Although not drained by a single vein, blood sampling from catheters in merging antecubital veins close to the forearm flexor muscles may yet closely reflect oxygen extraction (Hughson et al. 1996; Mostoufi‐Moab et al. 1998). Additionally, as arterial oxygen saturation and pulse oximetry is not found to be altered during forearm exercise, arteriovenous oxygen difference (a‐vO_2diff_) may be calculated (Raynaud et al. 1986; Hughson et al. 1996; Boushel et al. 1998). Ultimately, the forearm venous blood sampling may be combined with Doppler ultrasound measurements in the brachial artery to yield a compound experimental design for forearm $\dot{V}$O_2_ determination from rest to peak work rate (WR_peak_) (Nyberg et al. 2017).

Given the occupational relevance and putative clinical value of small muscle mass exercise, studies investigating forearm $\dot{V}$O_2_ dynamics, by experimental design combining Doppler ultrasound and venous blood gas, may provide important information of oxygen supply and demand in scenarios where central factors are not limiting. However, to the best of our knowledge, no studies have thoroughly tested the reliability of this compound method. As reliability of some measurements taken during exercise is found to decrease with increasing workload, the methods applicability approaching peak exercise may also be questioned (Bonaventura et al. 2015). Consequently, it remains unknown how precisely repeated measurements of various work rates (WRs) during dynamic handgrip exercise can be discriminated. Thus, the aim of this study was to evaluate this experimental design following exercise increments from rest to exhaustion. Specifically, given the high number of previous studies that have used this technique, we hypothesized that the reproducibility of $\dot{V}$O_2_ and its subcomponents, $\dot{Q}$ and a‐vO_2diff_, would be high and discriminate between the power increments of our exercise protocol (0.5 W), from light to heavy exercise.

## Methods

### Subjects

We recruited ten healthy, nonsmoking, moderately trained males to the current test–retest reliability study (Table 1). The subjects reported participation in various physical activities and sports 1–3 times per week, but with no specific training involving the forearms. All subjects included had a dominant right arm. The subjects’ overall training status was confirmed with a maximal oxygen consumption ($\dot{V}$O_2max_) test (Table 1). All subjects signed written informed consents, and the study was approved by the regional ethical committee, and carried out in accordance with the Helsinki declaration.

**Table 1:** Subject characteristics

Age (years)	24 ± 3
Body mass (kg)	78.7 ± 9.7
Height (cm)	179 ± 8
$\dot{V}$O_2max_	
L·min^−1^	4.48 ± 0.63
mL·kg^−1^·min^−1^	56.9 ± 4.8
Hemoglobin (g·100 mL^−1^)	14.7 ± 0.8
1RM (kg)	62 ± 10
Gross forearm volume (mL)	1.160 ± 0.178
Forearm flexor muscle mass (g)	500 ± 83

$\dot{V}$O_2max_, maximal oxygen uptake; 1RM, one repetition maximum.

Values are presented as mean ± SD.

### Study design

At the first test day, height, body mass, right forearm volume, and one repetition maximum (1RM) handgrip strength were measured. Additionally, the participants performed 15 min of low‐resistance dynamic handgrip exercise, to familiarize them with the exercise used the subsequent days. Subjects also performed a treadmill $\dot{V}$O_2max_ test.

The second and third test days were identical in form and content for the individual subject and consisted of measurements of forearm $\dot{Q}$, forearm venous blood gas, and systemic blood pressure at rest and at the end of each WR before increase during the incremental dynamic handgrip exercise to exhaustion. Subjects appeared in the laboratory at the same hour each day, separated by 48 h. They had received instructions of food and caffeine abstinence 3 h prior to testing. Subjects were also instructed to avoid exercise 24 h before tests.

### Maximal oxygen consumption

As a measurement of the participants’ overall training status, $\dot{V}$O_2max_ was measured with a Metamax II gas analyzer (Cortex Biophysik GmbH, Leipzig, Germany) following an incremental treadmill (Woodway PPS Med, Woodway USA, Inc., Waukesha) protocol. The test was initialized by a 15 min warm‐up period, followed by speed‐increments of 1 km·h^−1^ every minute at a fixed inclination of 5% until exhaustion. Criteria for achievement of $\dot{V}$O_2max_ were in accordance with established procedure (Wang et al. 2012).

### Anthropometric measurements

Forearm volume was measured by fluid‐displacement plethysmography (Boland and Adams 1996). Subcutaneous fat content was calculated from forearm length, circumference (O), and measurements of average skinfold thickness at three different locations on the forearm (S). Forearm flexor muscle volume was calculated specifically by multiplying gross forearm volume with factor 0.871 and forearm lean muscle volume with factor 0.53 to account for bone volume (Maughan et al. 1984) and the volume of active muscles (Sanchis‐Moysi et al. 2010), respectively. Forearm subcutaneous fat volume was subtracted, yielding forearm flexor muscle volume according to formula: {Forearm volume∙0.871 – [(S – 0.04)∙2^−1^]∙Length∙[(O_1_ + O_2_ + O_3_)∙3^−1^]}∙ 0.53. Muscle mass was calculated assuming a muscle tissue density of 1.049 g·cm^3^ (Radegran et al. 1999).

### Handgrip maximal strength and incremental exercise protocol

**Figure 1:**
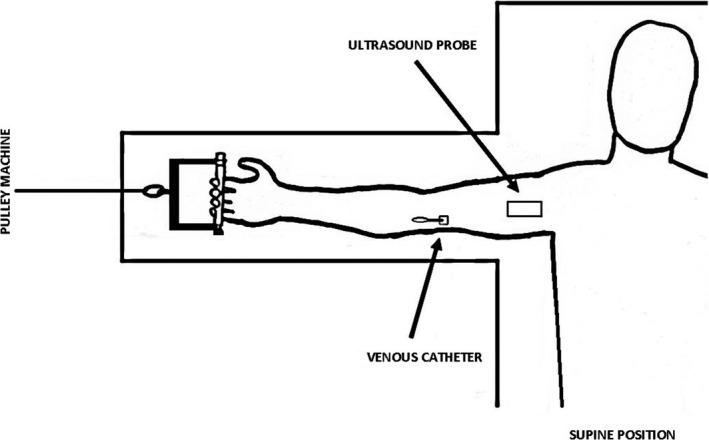
Custom‐made cable pulley with handgrip used for testing of forearm oxygen consumption ($\dot{V}$O_2_) during the incremental exercise protocol.

Dynamic handgrip measurements were collected utilizing a custom made handgrip device with the participants placed in a supine position with their right arm fully extended, abducted 90 degrees, at the level of the heart (Nyberg et al. 2017) (Fig. 1). The range of movement for the finger handgrip bar was 5.0 cm from a fully clenched fist to near full extension. The resistance was constant throughout the movement and adjustable at intervals of 0.25 kg by the use of a wire connected to a weight stack. After warming up with two sets with light to moderate load, the weight was gradually increased to obtain 1RM, defined as the heaviest load that the subject was able to move by handgrip contraction with a displacement of no less than 5 cm. 1RM was attained in all subjects within 4–6 attempts, after trials with 2.5–5.0 kg increments, separated by 3‐min rest periods.

Before the incremental exercise protocol, the subjects were kept in supine position in complete rest for 20 min, after which baseline measurements were made. During the subsequent handgrip exercise protocol, the subjects’ rhythmic muscle contractions had a fixed duty‐cycle of 1 sec contraction and 1 sec extension (0.5 Hz), guided by a metronome. From an initial WR of 1.0 W the WR was increased every 3rd minute by 0.5 W until failure. Failure was defined by one or more of the following: Discontinuation on the subject's initiative; inability to refrain from Valsalva maneuvers or excessive recruitment of muscles in the upper arm/torso > 10 sec despite verbal discouragement; inability to maintain the 5.0 cm range of movement or the 1 sec‐1 sec contraction/extension duty cycle >10 sec despite verbal encouragement.

### Hemodynamic measurements

Systemic blood pressure was measured continuously throughout the exercise protocol by beat by beat photoplethysmography (PortaPres, Finapres Medical Systems, Amsterdam, Netherlands) with inflatable cuffs on the 3rd and 4th finger on the left nonexercising hand. This arm was also put in a resting position along the body at the level of the heart. Each WR level yielded values of systolic blood pressure (SBP) and diastolic blood pressure (DBP). Mean arterial pressure (MAP) was calculated according to formula: MAP = (2∙DBP + SBP)∙3^−1^.

Measurements of blood velocity and intraarterial diameter of the brachial artery was measured with triplex mode Doppler ultrasound (Vivid E9, GE Healthcare, Little Chalfont) using a linear 4–12 MHz probe (GE 11L). The color‐Doppler function guided localization of the artery and avoidance of sampling from bifurcation sites. During the final 30 sec of each WR level a continuous 16 sec recording of blood velocity (spectral Doppler) and 2D B‐mode recordings were collected. Care was taken to ensure a clear image of the border between lumen and intima of the artery. Due to impact of muscle contractions, diameter measurements from four frames corresponding to contraction and four from relaxation phase were averaged for every subject in order to calculate diameter for each recording. Care was taken to adjust the scale of the spectral Doppler output to avoid aliasing, and the insonation angle was kept ≤60° to the axis of the vessel. Mean $\dot{Q}$, from each 16 sec recording, was calculated from vessel diameter (*D*_v_) and blood velocity (*V*_b_) according to formula: $\dot{Q}$ = π∙(*D*_v_ 2^−1^)^2^ *V*_b_ (Hughson et al. 1996).

### Blood gas

Prior to the exercise protocol, an 18G venous catheter (BD Venflon, Beckton Dickinson & Co., Franklin Lakes) was inserted by sterile procedure into a deep forearm vein of the right arm (Holling 1939; Mottram 1955). At rest, and during the final 30 sec of each of the 3‐min exercise increments, a 2 mL. venous blood sample was drawn from the catheter after a 2 mL. volume of spill‐blood. The samples were taken simultaneously as the corresponding Doppler‐ultrasound measurements. Immediately after being drawn, samples were gently mixed with dry heparine in syringes (Portex Line Draw Plus, Smiths Medical, St. Paul) and stored on ice. All samples were put through biochemical analysis within 30 min after being drawn using a RapidLab 1265 blood gas analyzer (Siemens Healthcare, Erlangen).

Venous lactate concentration, hemoglobin, venous partial oxygen pressure, and venous oxygen saturation were obtained from the blood samples. Arteriovenous oxygen difference (a‐vO_2diff_) was calculated from the following formula assuming 97% oxygen saturation in arterial blood: Blood oxygen (O_2_) concentration = 1.39∙hemoglobin∙(SatO_2_∙100^−1^) + 0.003∙pO_2_ (Barrett‐O'Keefe et al. 2014). Finally, $\dot{V}$O_2_ was calculated according to Fick's principle ($\dot{V}$O_2_ = $\dot{Q}$∙a‐vO_2diff_) and also expressed in relation to estimated mass of the exercising muscle when appropriate. Additionally, oxygen extraction was calculated by dividing a‐vO_2diff_ by the arterial oxygen concentration.

### Statistics

Statistical analyses were performed using IBM SPSS statistics 22.0, and figures produced with GraphPad Prism 5.0. For evaluation of test–retest agreement of arterial $\dot{Q}$, a‐vO_2diff_ and $\dot{V}$O_2_, a Bland–Altman analysis was used (Bland and Altman 1986). Coefficients of variation (CV) were determined as a percentage of the combined means of the test and retest values for each WR level. The least significant change (LSC) signified the percentage change for single‐subject 2‐point discrimination in time, with a 95% confidence interval, according to formula: LSC = Z (1.96)∙√2∙CV%_RMS_ (Kawalilak et al. 2015), where CV%_RMS_ is the combined CV for the number of test–retest comparisons performed. Correlations between variables were assessed with Pearson linear regression analysis. A mixed linear model was used to discriminate between mean values of measurements at baseline and across the different intensities of handgrip exercise. Absolute values for the respective variables were averaged from test and retest. All analyses performed were two‐tailed with a level of significance set to *P* < 0.05. A Bonferroni adjustment of the *P*‐value was applied when conducting multiple comparisons. For comparison of peak absolute values between test days a paired‐sample *t*‐test was used. Values are presented as mean ± standard deviation (SD) unless stated otherwise.

## Results

All participants successfully completed all testing procedures. The highest WR level evaluated in the present data (3.0 W) was equivalent to ~10% of the subjects’ 1RM.

### Forearm oxygen uptake and work rate

**Figure 2:**
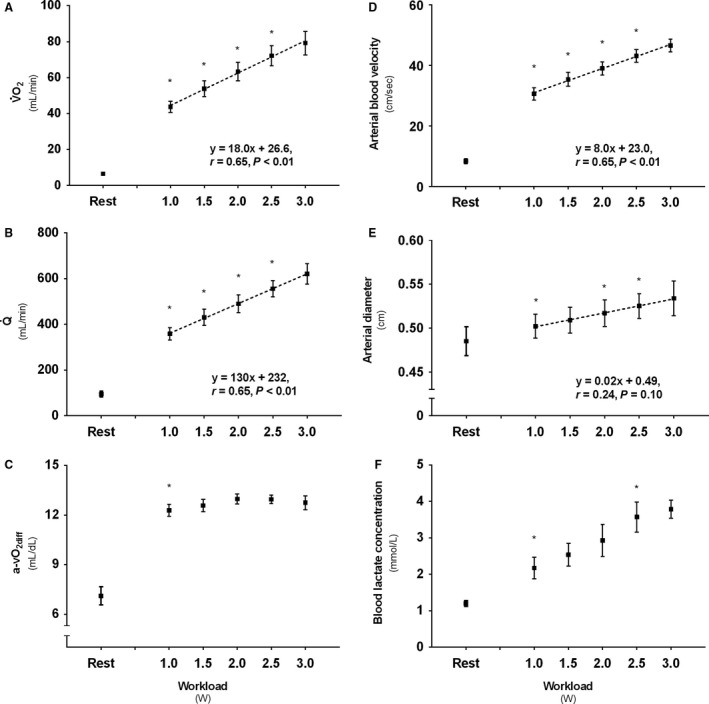
Descriptive values for (A) Forearm oxygen uptake ($\dot{V}$O_2_), (B) Brachial artery blood flow ($\dot{Q}$), (C) Arteriovenous oxygen difference (a‐vO_2diff_), (D) Brachial artery mean blood velocity, (E) Brachial artery diameter, and (F) Forearm venous lactate concentration following 3 min incremental steps of exercise intensity. * Denotes significant (*P* < 0.05) increase from previous condition. Boxes represent mean values from test and retest, with standard error of the mean (SEM).

$\dot{V}$O_2_ was 6.5 ± 2.5 mL·min^−1^ at rest, and exhibited a significant linear relationship with WR (Fig. 2A). The transition from rest to the first WR level (1.0 W) returned a higher $\dot{V}$O_2_ (*P* < 0.01), and $\dot{V}$O_2_ continued to increase for each of the subsequent single step increments from 1.0 to 2.5 W (*P* < 0.01). However, no significant increase was apparent from 2.5 W to the final WR level (3.0 W). Moreover, five of the subjects managed to complete one more increment (3.5 W), whereas three of these also were able to perform an additional final WR level (4.0 W). The various end‐points revealed a mean WR_peak_ of 3.4 ± 0.5 W. Peak oxygen uptake ($\dot{V}$O_2peak_) was 82 ± 34 mL·min^−1^, with a corresponding $\dot{Q}$ of 674 ± 277 mL·min^−1^, a‐vO_2diff_ of 12.5 ± 1.3 mL·dL^−1^ and venous pO_2_ of 3.6 ± 0.5 kPa. No significant differences were seen between test days for any of these variables, and consequently muscle diffusion capacity was also not different. If seen in relation to estimated forearm muscle mass, $\dot{V}$O_2_ versus WR yielded the same significant differences as the absolute values (1.3 ± 0.4 (Rest); 8.8 ± 1.4 (1.0 W); 10.7 ± 1.6 (1.5 W); 12.6 ± 1.8 (2.0 W); 14.4 ± 1.9 (2.5 W); 15.1 ± 2.0 (3.0 W); 15.7 ± 2.2 ($\dot{V}$O_2peak_) mL·100 g^−1^·min^−1^).

### Brachial artery blood flow

Resting $\dot{Q}$ was 95 ± 39 mL·min^−1^ and, as $\dot{V}$O_2_, exhibited a significant linear increase with rate of exercise (Fig. 2B), with single step increases from rest to 2.5 W (*P* < 0.01), but with no significant difference following the final 2.5–3.0 W increment. Muscle mass‐specific $\dot{Q}$ was 18.5 ± 5.5 (rest), 71.3 ± 8.5 (1.0 W), 85.3 ± 10.7 (1.5 W), 97.2 ± 11.1 (2.0 W), 111.3 ± 11.8 (2.5 W), 118.2 ± 11.4 (3.0 W), and 128.0 ± 15.6 (peak) mL·100 g^−1^·min^−1^, respectively.

### Arteriovenous oxygen difference

The a‐vO_2diff_ showed an increase from a resting value of 7.1 ± 1.7 mL·dL^−1^ to the first WR level (1.0 W; *P* < 0.05). No further significant changes in the a‐vO_2diff_ were observed following the subsequent WR increments (Fig. 2C). The concurrent oxygen extraction was 36 ± 9% at rest and increased to 61 ± 7% during the first WR level (1.0 W; *P* < 0.05).

### Blood velocity and brachial artery diameter

Blood velocity was 8.4 ± 2.5 cm·sec^−1^ at rest and showed a significant, linear, increase with increasing WR (Fig. 2D). The first WR level (1.0 W) was higher compared with rest (*P* < 0.05), and also increased following the three next (1.5 W; 2.0 W; 2.5 W) subsequent increments (*P* < 0.05). No further significant increase was evident from 2.5 W to the termination of exercise. Furthermore, all WR levels from 1.5 to 3.0 W showed a larger diameter than measured at rest (0.49 ± 0.05; *P* < 0.05). Significant increases were also evident in the transitions from 1.5 to 2.0 W and 2.0 to 2.5 W (*P* < 0.05) (Fig. 2E).

### Blood lactate concentration

Blood lactate concentration increased from rest (1.19 ± 0.25 mmol·L^−1^) to exercise at 1.0 W (*P* < 0.05), and also in the transition from 2.0 to 2.5 W (*P* < 0.05) (Fig. 2F).

### Vascular conductance and blood pressure

**Figure 3:**
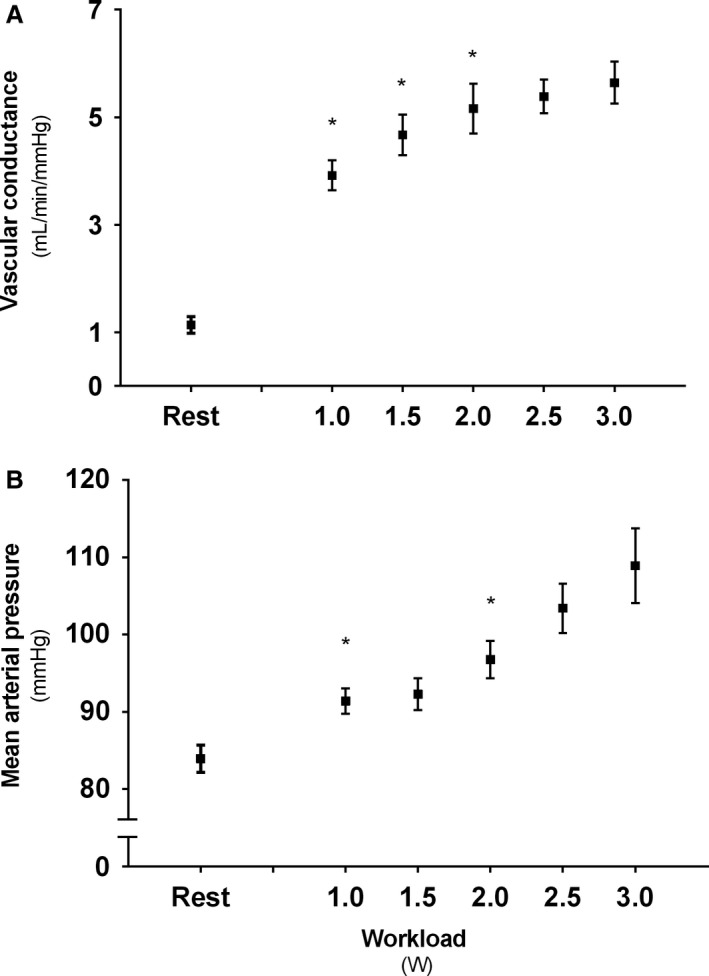
Descriptive values for (A) Forearm vascular conductance and (B) Mean arterial pressure (MAP) following 3 min incremental steps of exercise intensity. *Denotes significant (*P* < 0.05) increase from previous condition. Boxes represent mean values from test and retest, with standard error of the mean (SEM).

Forearm vascular conductance increased from rest to exercise at 1.0 W (*P* < 0.05), and all the WR levels were significantly higher compared to rest (*P* < 0.05). Increases in conductance were also evident in transitions 1.0–1.5 W and 1.5–2.0 W (*P* < 0.05), but no differences were apparent after 2.0 W (Fig. 3A). If seen in relation to estimated muscle mass, the vascular conductance yielded the same significant differences as observed for the absolute values, and was 2.2 ± 0.7 (rest), 7.8 ± 1.0 (1.0 W), 9.3 ± 1.3 (1.5 W), 10.1 ± 1.5 (2.0 W), 10.8 ± 1.4 (2.5 W), 9.5 ± 4.0 (3.0 W) mL·mmHg^−1^·min^−1^·100 g^−1^, respectively. MAP also showed an increase from rest to exercise (*P* < 0.05), and in the transition 1.5–2.0 W (Fig. 3B).

### $\dot{V}$O_2_‐$\dot{Q}$ relationship and test–retest reliability

**Figure 4:**
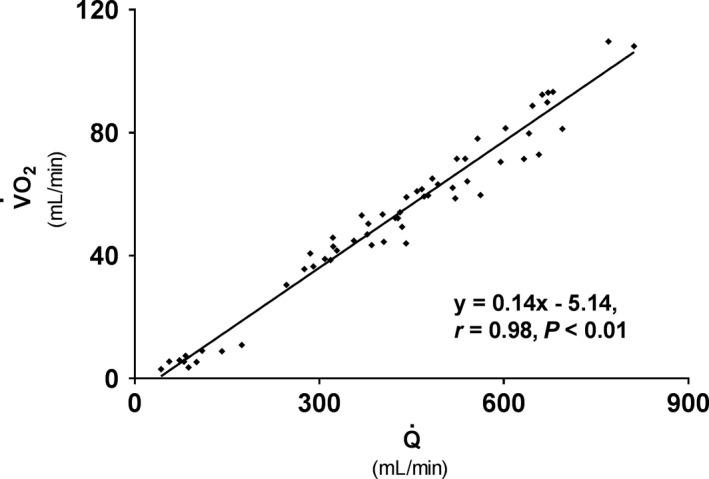
Linear relationship between brachial artery blood flow ($\dot{Q}$) and forearm oxygen uptake ($\dot{V}$O_2_) following dynamic handgrip exercise with 3 min (0.5 W) increments.

**Figure 5:**
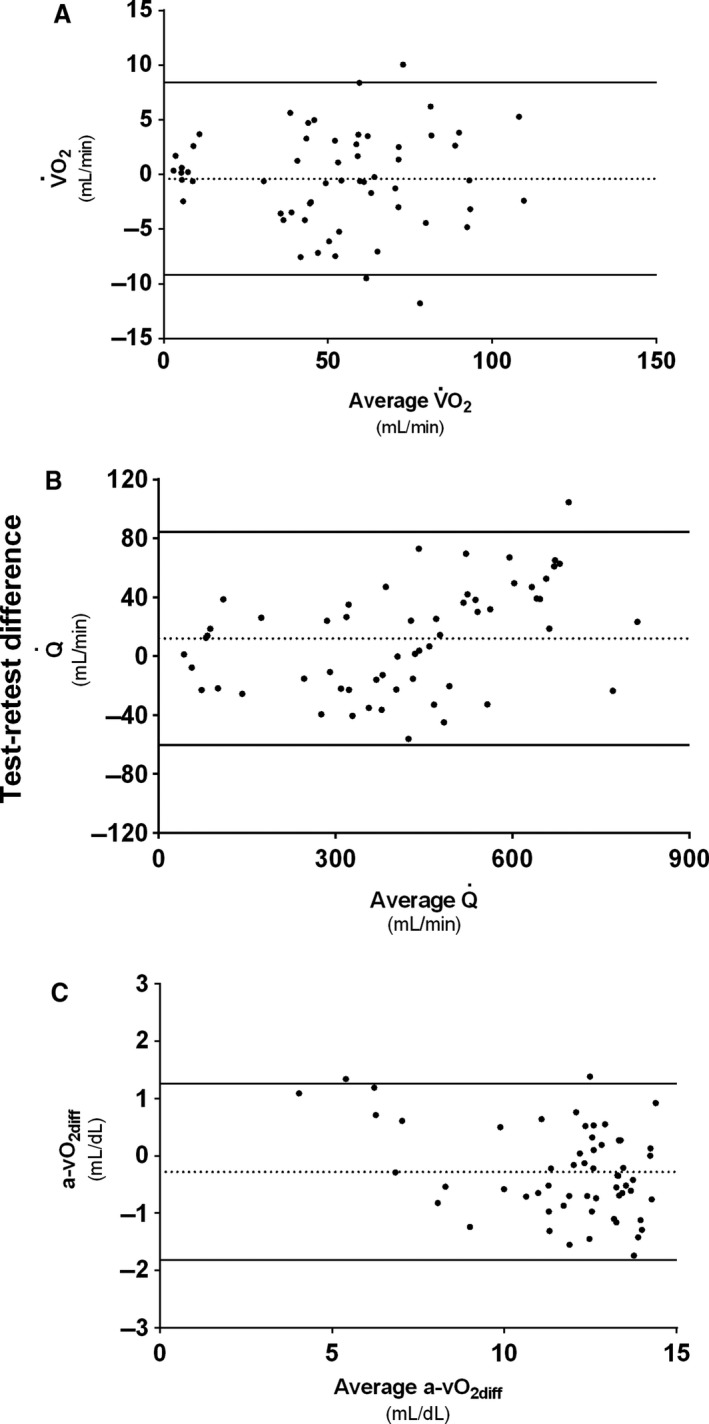
Bland–Altman analysis test–retest variability of (A) Forearm oxygen consumption ($\dot{V}$O_2_), (B) Brachial artery blood flow ($\dot{Q}$), and (C) Arteriovenous oxygen difference (a‐vO_2diff_). Mean test–retest differences are marked with dashed lines (···). Upper and lower 2SD limits of are marked with solid lines (―).

A close, significant, association between $\dot{V}$O_2_ and $\dot{Q}$ was observed following exercise (Fig. 4). Bland–Altman analyzes revealed that forearm $\dot{V}$O_2_ had a mean test–retest difference (−0.38 mL·min^−1^) that was not different from zero, with upper and lower 2SD limits of agreement of 8.39 and −9.15 mL·min^−1^, respectively (Fig. 5A). The CV was 19% at rest and 4–7% across WR levels (1.0–3.0 W) with no systematic change with increasing WR. The LSC was 16%. Test–retest values of $\dot{V}$O_2_ during different exercise intensities were not different when compared by the mixed linear model.

$\dot{Q}$ mean test–retest difference (11.9 mL·min^−1^) was not different from zero, with upper and lower 2SD limits of agreement of 84.1 and −60.4 mL·min^−1^, respectively (Fig. 5B). CV for $\dot{Q}$ measurements, as $\dot{V}$O_2_, ranged between 4 and 7% across WR levels with, again, no systematic change with increasing WR. The LSC was 16%. No significant differences were seen between test and retest values. Test–retest difference for measurements of arterial diameter was 0.00 cm with upper and lower 2SD limits of agreement of ±0.02 cm. CV was 1% for WR levels 1.0–1.5 W and 2% for WR levels 2.0–3.0 W. Test–retest difference for blood velocity measurements was 0.69 cm·sec^−1^ with upper and lower 2SD limits of agreement of 6.55 and −5.17 cm·sec^−1^, respectively. CV for blood velocity measurements ranged between 4 and 8% across WR levels, with no systematic change with increasing intensity. No significant test–retest difference in measurement was observed for either arterial diameter or arterial blood velocity.

Bland–Altman test–retest analyzes for a‐vO_2diff_ showed a mean difference (−0.28 mL·dL^−1^) not different from zero, with a 1.26 mL·dL^−1^ upper and a −1.82 mL·dL^−1^ lower 2SD limit of agreement (Fig. 5C). CV for a‐vO_2diff_ ranged from 3 to 5% across WR levels, with no systematic change with increasing WR. The LSC for calculated a‐vO_2diff_ by this method was 12%. A mixed linear model test–retest comparison of the values revealed no significant differences.

## Discussion

The handgrip exercise model has been extensively utilized to investigate occupationally relevant muscle metabolism when limitations to oxygen transport are not overshadowed by central factors. However, the reliability of what may be the most heralded method for forearm $\dot{V}$O_2_ determination, $\dot{Q}$ assessment in the brachial artery by Doppler ultrasound in conjunction with blood gas sampled in a deep forearm vein, has been uncertain. Thus, in this study we evaluated this compound approach, and the main findings were that (1) Forearm $\dot{V}$O_2_ increased linearly with the rate of exercise, with distinguishable WR increments of 0.5 W (2) $\dot{V}$O_2_ and its subcomponents, $\dot{Q}$ and a‐vO_2diff,_ all demonstrated consistent good test–retest reliability following all WR levels. Taken together, these findings demonstrate that Doppler ultrasound measurements in the brachial artery, in conjunction with forearm venous blood sampling, is an excellent and applicable approach for forearm $\dot{V}$O_2_ determination during dynamic handgrip exercise with increasing intensity.

### Oxygen uptake, blood flow, arteriovenous oxygen difference, and rate of exercise

This study documented a linear relationship between $\dot{V}$O_2_ and WR with values that were in accordance with what has previously been documented (Hartling et al. 1989; Hughson et al. 1996; Wilkins et al. 2008). The $\dot{V}$O_2_‐WR relationship was also similar to what has been demonstrated in the isolated quadriceps (Saltin 1985; Richardson et al. 1993), and there was a very close association between the increase in $\dot{V}$O_2_ and brachial artery $\dot{Q}$, with ~7.0 mL·min^−1^ $\dot{Q}$ mL^−1^·min^−1^ $\dot{V}$O_2_. This is within, but in the high end, of the range typically documented for this relationship (Saltin and Calbet 2006). However, forearm exercise appears to yield a somewhat higher $\dot{Q}$ per $\dot{V}$O_2_ compared to lower extremity exercise (Hughson et al. 1996; Van Beekvelt et al. 2001), and this may be explained by the poor oxygen extraction seen in arms (Calbet et al. 2005). Of note, the final increment in the protocol in this study did not elicit a significantly higher $\dot{V}$O_2_. As half of the subjects failed to continue the exercise beyond this WR level, this may, in accordance with previous observations (Nyberg et al. 2017), be an indication of a $\dot{V}$O_2_ plateau. The lactate concentration in the failing subjects’ blood (4.6 ± 1.1 mmol·L^−1^) also indicated that they were at the point, or close to the point, of exhaustion compared to the other participants (3.3 ± 0.3 mmol·L^−1^).

The greatest uncertainty with the compound Doppler‐ultrasound/catheterization method is the venous oxygen content (Hughson et al. 1996). It is difficult to ensure that the catheter is localized in the vein that is most representative for draining the exercising musculature, because in contrast to, for example, leg exercise, there is no major single outflow vein. However, in previous studies (Hughson et al. 1996; Van Beekvelt et al. 2001; Nyberg et al. 2017), as well as this study, changes in the rate of exercise and arm position appeared to be reflected in alterations in oxygen extraction. To control for all venous outflow, the subclavian vein could have been an alternative catheter placement. Although this may have been preferable to detect differences in venous oxygen content before and after training or when comparing different groups, it may have yielded lower absolute values because of the mixing with blood from nonactive tissue (Berg et al. 2017). Nevertheless, the direct venous sampling appears to offer some benefits over other experimental designs with regards to absolute forearm a‐vO_2diff_, and consequently $\dot{V}$O_2_, determination. The noninvasive near infrared spectroscopy (NIRS) technique, for example, appears to underestimate $\dot{V}$O_2_ (Hicks et al. 1999; Gurley et al. 2012). Although the experimental design in this study is invasive, a criticism against it could be that it is not invasive enough. Compared to the indicator dilution methods it lacks an arterial catheter for measurements of oxygen content. However, given the very small exercising muscle mass, arterial oxygen saturation has been found not to change with handgrip exercise (SaO_2_ 97 ± 1%) (Boushel et al. 1998), and it is reasonable to advocate avoidance of this additional invasive procedure. Thus, the method evaluated in this study may be an appropriate balance between an invasive and noninvasive approach to assess occupationally relevant forearm muscle metabolism.

Utilizing the method in the current investigation, a recent study (Nyberg et al. 2017) observed that the a‐vO_2diff_ remained constant from moderate to high intensity, implying that a higher $\dot{Q}$ would be necessary to meet the oxygen demand. Again, in this study, the a‐vO_2diff_ appeared to remain fairly constant after the initial rapid increase from rest to the first WR level. This is in stark contrast to what has been documented for, example, the quadriceps, where oxygen extraction is shown to continuously increase up to levels around 85% (Richardson et al. 1993). Not only the type of muscle group and mass, but also different training status may affect this divergence (Gifford et al. 2016). If seen in relation to estimated forearm muscle mass, the $\dot{V}$O_2_‐WR and $\dot{V}$O_2_‐$\dot{Q}$ slopes confirmed the linear relationships with a peak perfusion and $\dot{V}$O_2peak_ of ~128 ± 16 and ~16 ± 5 mL·100 g^−1^·min^−1^, respectively. Recognizing that this is very low compared with, for example, the quadriceps (Richardson et al. 1993) it is of importance that $\dot{Q}$, and thus $\dot{V}$O_2_, is largely dictated by muscle contractions, and that peak perfusion is about threefold higher during the relaxation phase (Nyberg et al. 2017).

Our data showed that the brachial arterial diameter contributed to the linear increase in $\dot{Q}$, and $\dot{V}$O_2_. The dynamic behavior of the conduit artery vessel with WR is in accordance with existing literature (Shoemaker et al. 1997), and underpins the importance of not determining $\dot{Q}$ based on a constant diameter measured at rest. The arm position was at the level of the heart in our study. However, arm position is previously shown to not influence the diameter response (Shoemaker et al. 1997), implying that muscle metabolism may play an important role in the conduit artery regulation. On the contrary, recognizing that blood velocity is largely influenced by the arm positioning (Hughson et al. 1996; Shoemaker et al. 1997), this factor should certainly be taken into consideration when comparing $\dot{Q}$ between the various exercise protocols. In this study, the blood velocity response was assisted by a large increase in MAP, typical for arm exercise (Calbet et al. 2015). If the arm had been placed lower, this would have enhanced the MAP response (Shoemaker et al. 1997). Moreover, the vascular conductance increased in a similar curvelinear fashion as what previously has been observed for both arm and leg exercise (Calbet et al. 2015), with a somewhat steeper slope for the first WR increments compared to the later steps.

### Test–retest reliability

Our data revealed that the compound Doppler ultrasound/venous catheterization method had a $\dot{V}$O_2_ test–retest reliability with 2SD limits of agreement that implied it was able to detect differences between the submaximal increments of 0.5 W in the exercise protocol. The CV ranged between 4 and 7% and, importantly, with no systematic variation relative to the WR. Of notice, the lower resting values mathematically returned a higher CV (19%). The $\dot{V}$O_2_ in this study was calculated from the Fick principle, and consequently a result of both Doppler ultrasound and venous blood gas measurements. Both these subcomponents independently demonstrated a good test–retest reliability. A well‐known challenge with the Doppler ultrasound technology has been difficulties with movement artifacts (Casey et al. 2008). However, with advancing technology, and facilitated by the application of a very small muscle mass, this was not recognized in this study, and the CV was 4–7% with no apparent tendency to increase with the rate of exercise. Each WR increment induced about a 60–70 mL·min^−1^ elevation in $\dot{Q}$, which implies that the method was borderline for detecting the 0.5 Watt exercise differences. Although the B‐mode recordings of arterial diameter and arterial blood velocity ensured mutual temporal and anatomical proximity, they represent two independent sources of error. While the diameter, in accordance with previous literature (Radegran 1997) had a CV of 1–2%, the blood velocity showed a CV of 4–8% following the increasing rate of exercise. This should both be considered satisfying, especially given the complex and dynamic blood velocity behavior observed during muscle contractions (Robergs et al. 1997).

Our data returned a good a‐vO_2diff_ reliability with the CV stable at <7% with increasing intensity, and 2SD limits of agreement implying that the method was able to detect differences >1.8 mL·dL^−1^. In comparison, the transition from rest to exercise resulted in a ~5 mL·dL^−1^ difference. In accordance with a previous study (Nyberg et al. 2017), no further elevation of the a‐vO_2diff_ was seen with increasing rate of exercise. Of importance, for reliable measurements of the a‐vO_2diff_, care was taken to ensure a similar catheter placement and blood sample timing. However, one factor that would likely further improve the measurement of venous oxygen content is repeated sampling for each time point. This should be accompanied with simultaneous measurements of $\dot{Q}$. Of the elements that have the potential to improve this compound method, this may be one of the simplest, yet effective, actions that should be considered. Finally, of importance, the reliability of this compound method will depend on whether experiments are carried out in the same laboratory, by the same technician, and the equipment used.

## Conclusion

Forearm $\dot{V}$O_2_ assessment by Doppler ultrasound and venous blood gas is a minimally invasive approach to investigate small muscle mass metabolism in occupationally relevant musculature. This study provides, for the first time, a thorough evaluation of this compound method during exercise and revealed that its components increased linearly with increasing work and had a very good reliability able to discriminate between power increments of 0.5 W from light to heavy work loads. These results advocate the utilization of the method in future research.

## Conflict of Interest

The authors declare no conflicts of interest, financial or otherwise.
